# ASO Author Reflections: Towards Fluorescence Guided Tumor Identification for Precision Breast Conserving Surgery

**DOI:** 10.1245/s10434-021-10626-6

**Published:** 2021-08-18

**Authors:** Maria Leiloglou, Martha S. Kedrzycki, Daniel S. Elson, Daniel R. Leff

**Affiliations:** 1 Hamlyn Centre, Institute of Global Health Innovation, Imperial College London, South Kensington Campus, London, UK; 2 Department of Surgery and Cancer, Imperial College London, London, UK; 3 Department of Breast Surgery, Charing Cross Hospital, Imperial College Healthcare NHS Trust, London, UK

## Past

Breast conserving surgery (BCS) is the cornerstone surgical treatment in breast cancer; however, it is associated with a risk of reoperative intervention due to positive resection margins.^1^ Reoperation puts patients at risk of perioperative complications including infection and inferior cosmetic outcomes, as well as delays to adjuvant treatment. From the payor perspective, reoperation creates financial toxicity due to the direct costs of intervention as well as indirect costs due to time off work. Conventional localization techniques only provide approximate tumor location and fail to provide tumor identification or tissue characterization.^1^ Tumor identification through fluorescence guided surgery (FGS) is Food and Drug Administration approved for cancers such as glioblastoma where negative margins are paramount,^2^ and these benefits could be applied to BCS through demarcating tumor location, size, and invasiveness.

## Present

A prospective clinical trial was conducted using an in-house fluorescence camera system in 40 patients undergoing BCS. Indocyanine green (ICG) at 0.25 mg/kg was administered to 20 patients at the start of the operation, and 20 patients intraoperatively, once skin flaps were raised. The patients who received an intraoperative ICG injection demonstrated a stronger tumor background ratio (TBR) than those who received preoperative ICG (3.18 ± 1.74 vs 2.10 ± 0.92, *p* = 0.023). However, sensitivity and specificity for the intraoperative cohort was not statistically different from that of the preoperative cohort (0.82:0.93 vs 0.66:0.90 respectively, *p =* 1.105: *p =* 0.909).^3^ The key finding of this study is relative improvement in TBR with intraoperative fluorescence injection. Whilst ICG provides a clinically adequate TBR (> 1.5),^3^ further work is required to improve the diagnostic accuracy for the clinical adoption of fluorescence guided breast surgery.

## Future

Although when compared with previous specimen-based analysis, pixel-based image analysis was identified to be superior^4^,^5^ and high accuracy was attained, BCS using ICG requires appropriately powered clinical trials to determine whether patient outcomes are improved. Future work should also focus on improving results either through camera system modifications, enhancing image processing, and/or using targeted fluorophores specific to breast cancer towards clinical adoption of fluorescence guided breast surgery.

## References

[1] Ananthakrishnan P, Balci FL, Crowe JP (2012). Optimizing surgical margins in breast conservation. Int J Surg Oncol.

[2] Eljamel S (2015). 5-ALA fluorescence image guided resection of glioblastoma multiforme: a meta-analysis of the literature. Int J Mol Sci.

[3] Kedrzycki MS, Leiloglou M, Chalau V, Chiarini N, Thiruchelvam PTR, Hadjiminas DJ (2021). The impact of temporal variation in indocyanine green administration on tumor identification during fluorescence guided breast surgery. Ann Surg Oncol.

[4] Pop CF, Barbieux R, Moreau M, Noterman D, Neubourg FD, Chintinne M (2019). Tumor localization and surgical margins identification in breast cancer using ICG-fluorescence imaging. Eur J Surg Oncol.

[5] Veys I, Pop C, Barbieux R, Moreau M, Noterman D, Neubourg FD (2018). ICG fluorescence imaging as a new tool for optimization of pathological evaluation in breast cancer tumors after neoadjuvant chemotherapy. PLoS ONE.

